# 

**Published:** 1884-07

**Authors:** 


### CONTENTS.

ORIGINAL COMMUNICATIONS: PAGE.

I. Physical Conditions Essential to the Production of Tympanitic Resonance, and Pathological States of Pulmonary Tissues in which it occurs. By R. H. Babcock, M. D., Chicago ..... 1

II. Our Children's Eyes and Books. By F. C. Hotz, M. D., Chicago ..... 10

III. Surgical Treatment of Acute, Circumscribed, Pulmonary Gangrene; Second Successful Operation. By Professor C. Fenger, M. D. ..... 19

IV. A Case of Aneurism of the Abdominal Aorta, with Notes of Post Mortem Examination. By Mark H. Sears, M. D., Leadville, Col ..... 22

V. Injections of Glycerine and Carbolic Acid for the Preservation of Bodies for Dissection. O. T. Freer, M. D., Munich ..... 27

VI. Surgical Delusions. An Abstract of the Address in Surgery of the Medical Society of the State of Pennsylvania for 1884. By John B. Roberts, M. D., Professor of Anatomy and Surgery in the Philadelphia Polyclinic ..... 29

In Memoriam, Samuel David Gross ..... 35

EDITORIAL:

More About Public Laundries ..... 36

Anatomical Bill ..... 38

DOMESTIC CORRESPONDENCE ..... 41

SOCIETY PROCEEDINGS ..... 46

SELECTIONS:

Gun-Shot Wounds of the Small Intestines. By Charles T. Parkes, M. D., Professor of Anatomy in Rush Medical College, Chicago, Ill ..... 59

ASSOCIATION PROCEEDINGS ..... 94

BOOK REVIEWS ..... 95

